# Supplementary material for: MeQTL Mapping in African American Hepatocytes Reveals Shared Genetic Regulators of DNA Methylation and Gene Expression
Source: bioRxiv. 2025 Jan 26:2025.01.23.634506. Preprint. [Version 1] doi: 10.1101/2025.01.23.634506 (PMC11785176; doi:10.1101/2025.01.23.634506)
Supplement: Supplement 3 [file NIHPP2025.01.23.634506v1-supplement-3.pdf]

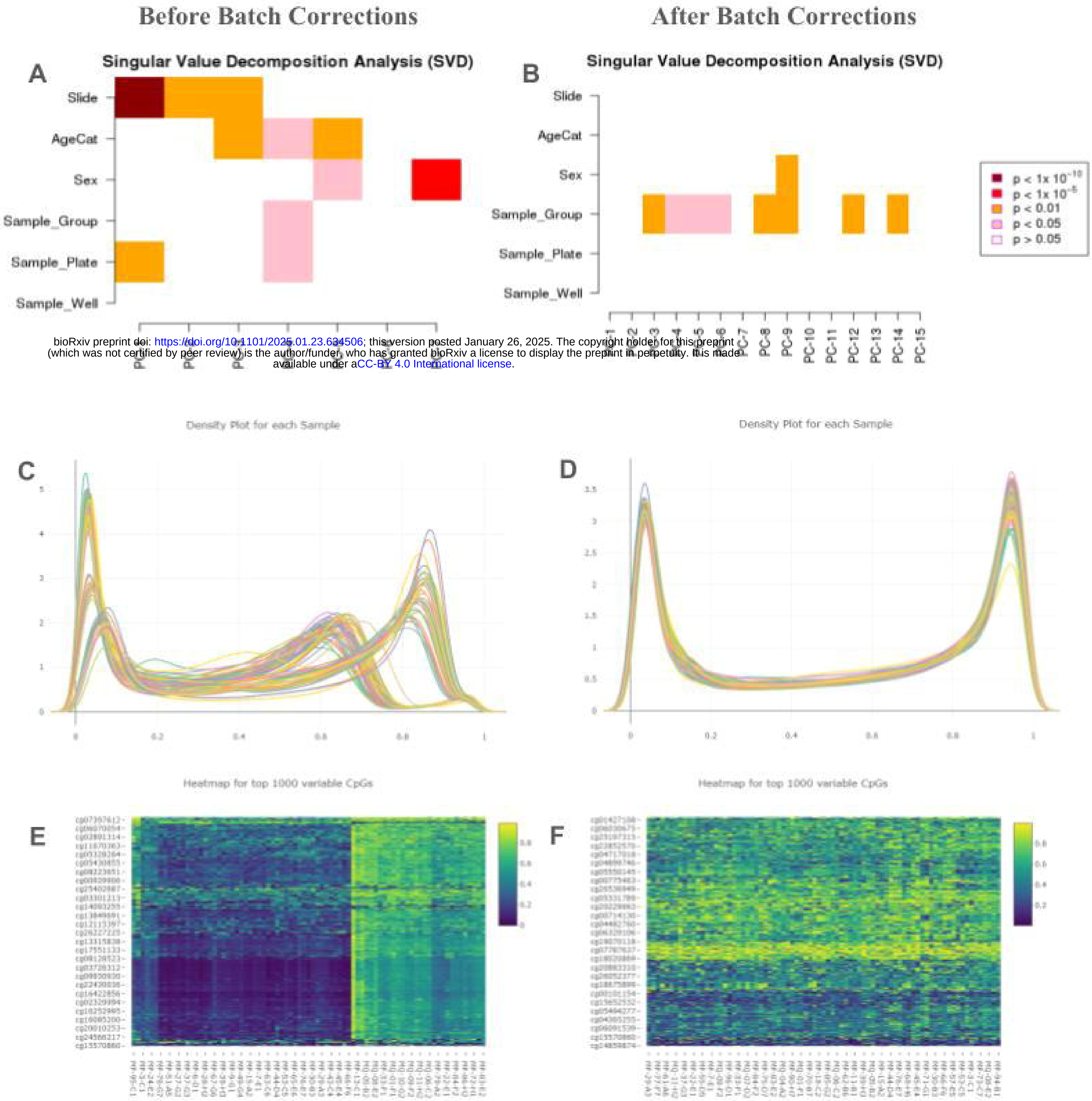

bioRxiv preprint doi: https://doi.org/10.1101/2025.01.23.634506; this version posted January 26, 2025. The copyright holder for this preprint (which was not certified by peer review) is the author/funder, who has granted bioRxiv a license to display the preprint in perpetuity. It is made available under aCC-BY 4.0 International license.

Supplementary Figure 1: Reduction of batch effects using the *ChAMP* package in R. a, b) Use of the ChAMP.runCombat function reduces the effects of technical variation, such as slide and sample plate, on methylation measurements. c, d) Prior to normalization, methylation measurements fall in three clusters on the density plot. Each cluster represents a separate sequencing batch. Following normalization and corrections for batch effects, the distributions for each batch align. e, f) Prior to normalization, variability in methylation at the top CpGs is primarily driven by batch effects, as there are strong differences between the clusters of samples. Following normalization, all samples display similar variability at these CpGs. These three figures demonstrate that *ChAMP* sufficiently corrected for batch effects to reduce the effects of technical variation on methylation measurements.
